# Assessing Endovenous Heat-Induced Thrombosis in Flush Endovenous Laser Ablation: A Study on Incidence, Risk Factors, and Patient Outcomes

**DOI:** 10.3390/jcm14176165

**Published:** 2025-08-31

**Authors:** Mihai Cosmin Burta, Adela Avram, Radu Florian Avram, Steven Kristofor Rogers, Frank Lee Bowling, Stefan Ionac, Mihai Edmond Ionac

**Affiliations:** 1 Faculty of Medicine, Victor Babes University of Medicine and Pharmacy, 300041 Timișoara, Romania; mihai.burta@student.umft.ro (M.C.B.); stefan.ionac@umft.ro (S.I.); 2 ArTerra Health Clinic, 300402 Timișoara, Romania; adelas51@yahoo.com (A.A.); rdk_avram@yahoo.com (R.F.A.); 3 Manchester Academic Vascular Research and Innovation Centre (MAVRIC), Manchester Vascular Centre, Manchester University NHS Foundation Trust, Oxford Road, Manchester M13 9WL, UKfrank.bowling@manchester.ac.uk (F.L.B.); 4 Division of Cardiovascular Sciences, School of Medical Sciences, Faculty of Medicine, Biology and Health, University of Manchester, Manchester M13 9PT, UK; 5 Division of Nursing, Midwifery and Social Work, School of Health Sciences, Faculty of Medicine, Biology and Health, University of Manchester, Manchester M13 9PT, UK; 6 Division of Diabetes, Endocrinology and Gastrointestinal Medicine, School of Medical Sciences, Faculty of Medicine, Biology and Health, University of Manchester, Manchester M13 9PT, UK

**Keywords:** chronic venous insufficiency, endovenous heat-induced thrombosis, EHIT, CEAP, fEVLA

## Abstract

**Introduction:** The introduction of radial-firing laser fibers has minimized catheter-to-vein distance during endovenous laser ablation (EVLA) for the great saphenous vein (GSV) and anterior saphenous veins (ASVs). This study investigates flush endovenous laser ablation (fEVLA) effectiveness in addressing chronic superficial venous insufficiency (CVI). **Materials and methods:** This single-center retrospective study analyzed consecutive fEVLA cases with duplex ultrasound follow-up at 1, 4, and 12 weeks. The primary endpoint was clinically significant endovenous heat-induced thrombosis (classes III–IV). **Results:** Three hundred and twelve patients were recruited (405 affected limbs, 369 GSV, and 36 ASV). CEAP classifications were stratified as follows: C2 in 6.1%, C3 in 34.2%, C4 in 44%, C5 in 2.7%, and C6 in 12.7% of cases. Perforator ligation, phlebectomy, or foam sclerotherapy were carried out in conjunction with EVLA. fEVLA was feasible in all cases. The success rate was 96.78%, defined as EHIT classes 1 and 2, and assessed by ultrasound one week postoperatively. Clinically significant EHIT (2.5% class 3 and 0.2% class 4) was managed with 15 mg rivaroxaban twice daily for 21 days. Follow-up at 4 weeks revealed complete resolution of all EHIT 3–4 cases. No cases of pulmonary embolism or deep vein thrombosis were observed during the study or follow-up period. **Conclusions:** fEVLA is a safe treatment for superficial CVI across various CEAP classes, and with prompt detection, the minimal complication rate can be completely resolved.

## 1. Introduction

Both high ligation and stripping (HL/S) and endovenous laser ablation (EVLA) have shown broadly comparable long-term outcomes for primary varicose veins, although patterns and rates of recurrence depend on device characteristics, fiber design, and procedural technique [1,2,3,4]. With current 1470 nm systems and radial fibers, mid- to long-term truncal occlusion is typically around 90–95%, and meta-analytic five-year SFJ recurrent reflux after EVLA is about 22% (95% CI 14–32%) [2,5,6].

Unlike HL/S—where neovascularization in the groin frequently contributes to recurrence—EVLA-related recurrence is more often driven by a refluxing residual (remnant) stump at the saphenofemoral junction (SFJ), rather than true neovascularization [7]. In clinical follow-up, a common pattern is antegrade propagation of reflux from the SFJ into the anterior saphenous vein (ASV) [8], which can sustain or re-establish varicosities even after an initially successful truncal ablation [7].

Traditionally, EVLA was performed with ablation terminating 1–2 cm distal to the SFJ to reduce the risk of endovenous heat-induced thrombosis (EHIT) (Table 1)—a thrombotic extension from the treated superficial trunk into the common femoral vein (CFV) [9].

With contemporary radial fibers, energy delivery is circumferential and more homogeneous than with bare-tip fibers, enabling precise ultrasound-guided tip placement at the SFJ heel under adequate tumescence [10,11]. These advances do not, by themselves, mandate a shorter untreated segment; rather, they allow experienced teams to deliberately target a minimal residual stump (~0–2 mm) while maintaining a low risk of clinically significant EHIT [12]. Observational and comparative data report feasibility, lower stump-related reflux, and reassuring junctional safety with flush endovenous laser ablation (fEVLA) [6,7,13,14]. At the same time, an RCT found no significant reduction in ASV reflux at 24 months with flush versus standard positioning, indicating that benefits are anatomy- and technique-dependent [15].

This evolving approach—fEVLA—seeks to minimize the residual stump and, by extension, the likelihood of junction-related reflux into the ASV over time [7,12]. In this investigation, we analyzed both minor and major complications associated with fEVLA to evaluate its overall effectiveness and practicality in routine practice. The primary objective was to assess the occurrence of clinically significant EHIT (i.e., classes 3 and 4), while secondary outcomes included procedure-related complications (particularly DVT and PE), anatomic success, technical feasibility, and rates of flush occlusion at predefined follow-up intervals [9].

## 2. Materials and Methods

This retrospective, observational, single-center study was carried out in a secondary-referral private vascular clinic and encompassed fEVLA procedures performed consecutively between October 2022 and June 2024. This study was aligned with contemporary quality-improvement recommendations for superficial venous interventions and European guidance for chronic venous disease; eligibility required a concordance between symptoms and duplex-demonstrated superficial axial reflux, while exclusions were limited strictly to absence of the scheduled follow-up ultrasound assessments at 1, 4, or 12 weeks, thereby preserving an unselected cohort reflective of routine practice under standardized imaging and reporting. Ethics approval was granted by the institutional review board (protocol No. 97/03.10.2022 rev 2024), and written informed consent was obtained from all participants [16,17,18,19].

Detailed demographics on sex, age, Body Mass Index (BMI), allergies, medical history, clinical manifestations, and previous medication, interventions, and physical examination findings were documented in addition to imaging findings. Duplex ultrasound (DUS) examination was performed using a GE Vivi^TM^ T8 machine with an 8–12 MHz linear transducer, with the patient in a standing position. The imaging protocol included visualization of the saphenofemoral junction, measurement of vein diameter in multiple locations, localization and diameter of perforators, and pinpoint reflux assessment and duration. Pathological valve function at the saphenofemoral junction was assessed by instructing the patient to perform the Valsalva and/or augmenting maneuvers (manual calf compression), while color and spectral Doppler imaging was used to quantify the resulting hemodynamic changes. Patients with CEAP clinical classification ranging C2 to C6, presenting symptoms and reflux over 0.5 s, were considered for fEVLA treatment.

Informed consent was obtained pre-procedurally by the operating surgeon, ensuring all patients were fully informed and agreed to the treatment plan. The fEVLA procedure followed an ambulatory, walk-in–walk-out protocol.

In this study, flush EVLA was defined anatomically, namely ablation initiated with the radial fiber tip positioned at the SFJ heel under real-time ultrasound, under adequate tumescence, such that the residual superficial venous stump measured ≤2 mm from the confluence with the CFV both on immediate intraoperative assessment and at the 1-week duplex control [7,9,12].

In limbs with concomitant ASV incompetence, we used a two-access, single-session approach: the GSV was cannulated and ablated flush at the SFJ, with the radial fiber tip positioned at the SFJ heel under ultrasound to achieve a residual stump ≤ 2 mm, and, through a separate puncture, the ASV was cannulated and the fiber advanced cranially to its ostium where flush ablation was performed at the ASV–GSV junction; this protocol therefore produced true SFJ flush closure and ASV flush closure in the same sitting, and such limbs were recorded as fEVLA at SFJ with concomitant ASV ablation (predefined subgroup for analysis), whereas only limbs in which the GSV trunk was intentionally left untreated were labelled ASV-only flush.

Although flush positioning of the fiber tip at SFJ is not explicitly mandated in manufacturer guidelines, it is not contraindicated either. According to the Instructions for Use (IFU) of the VENEX 360° laser fiber (KLS Martin, Jacksonville, FL, USA, REF 79-350-00-04, Revision 04, 2023), the radiopaque glass-dome tip of the radial fiber was advanced beyond the distal edge of the sheath and positioned precisely at the SFJ heel under continuous ultrasound guidance, with a deliberately maintained visible clearance between the heated dome and the silicone/plastic sheath in order to prevent thermal softening or deformation of the introducer and to avoid any heat shadowing at the junction; after tumescent infiltration, both tip position and sheath clearance were re-verified in longitudinal and transverse planes to exclude inadvertent cannulation of the superficial epigastric vein or another tributary, and energy delivery commenced only after reconfirmation of a stable, flush, sheath-cleared configuration, with minor adjustments (external compression or redistribution of tumescence) made whenever alignment drifted, as per the IFU. In our protocol, all procedures were performed in strict adherence to these recommendations, using real-time ultrasound guidance to ensure correct positioning at the SFJ, with adjustments performed as needed using external compression or tumescence to optimize fiber alignment. All patients were fully informed regarding the procedural technique, including flush positioning, and provided written informed consent prior to the intervention. This study received institutional ethical approval (No. 97/03.10.2022 rev 2024), and no deviations from the device’s intended use or IFU were made throughout this study.

All procedures were performed by three vascular surgeons with expertise in endovenous interventions, following the same standardized protocol for flush fiber placement and intraoperative monitoring.

**Figure 1:**
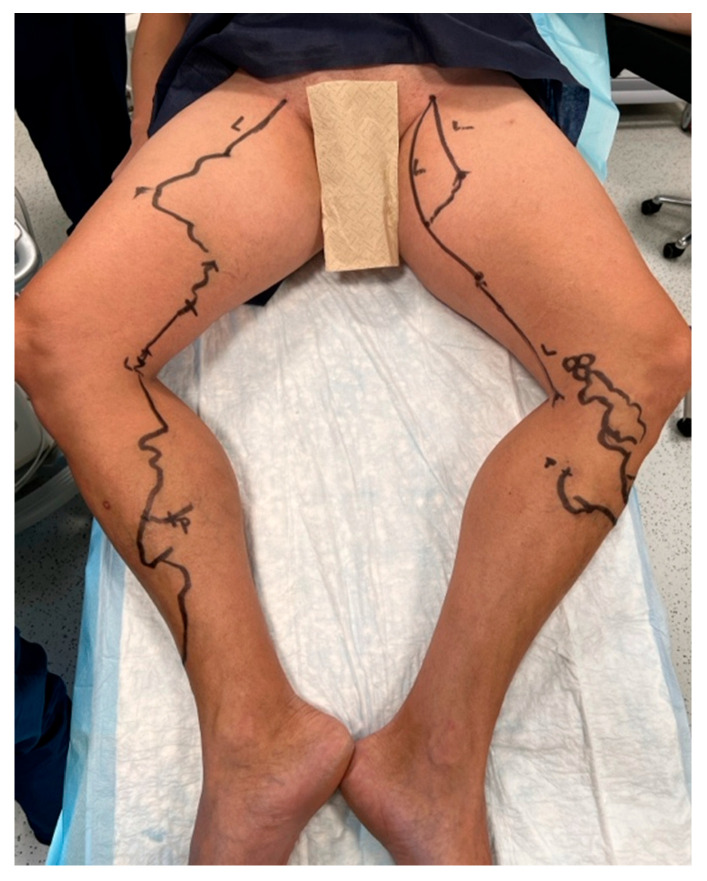
Preoperative markings of vein routes, puncture sites, and microphlebectomy sites.

**Figure 2:**
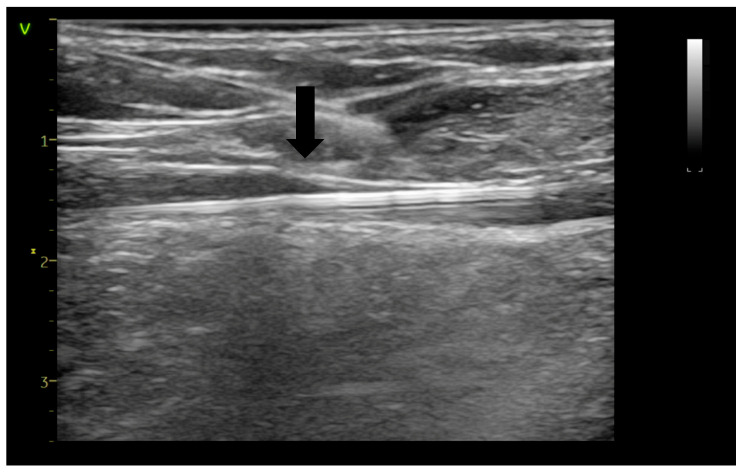
Ultrasound-guided tumescent anesthesia applied in the saphenous compartment (arrow—anesthesia needle tip).

**Figure 3:**
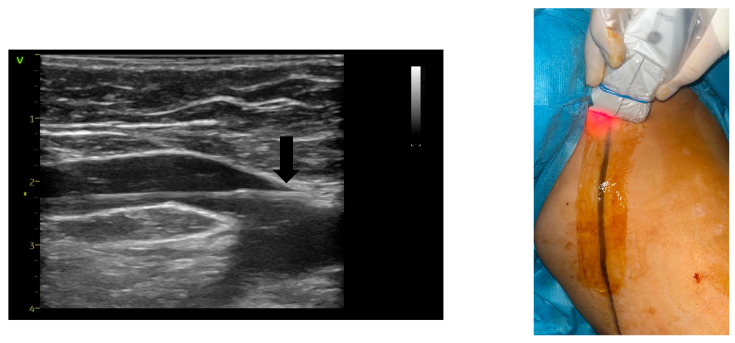
Catheter tip (arrow) positioning at the saphenofemoral junction using ultrasound guidance (**left**) and pilot setting guidance (**right**).

Initial steps included preoperative ultrasound-guided markings of vein routes, reflux points, and planned microphlebectomy sites if indicated (Figure 1). Sedation was achieved using a combination of remifentanil and propofol. In combination with local anesthesia, this protocol prevents the patient from experiencing any discomfort or pain during the intervention, especially if the laser ablation is completed by phlebectomies, allowing her/him to have an almost instantaneous recovery. The KSL Martin VENEX 360° laser fiber, connected to a KSL Martin Diomax 1550 nm source, was introduced through a radial sheath (Merit Medical Prelude^®^ 6F Sheath Introducer, South Jordan, UT, USA) inside the targeted veins (GSV, ASV). Ultrasound-guided local tumescence (500 mL of 0.9% sodium chloride, 500 mg of lidocaine, 1 mg of adrenaline, and 20 mL of sodium bicarbonate at a concentration of 84 mg/mL) was applied in the fascial saphenous compartment under direct visualization using GE Venue Go^TM^, with an 8–20 MHz linear probe and peristaltic pump Nouvag AG DP 30 for enhanced precision, optimal vein compression, effective ablation, and patient comfort (Figure 2). The tip of the catheter was precisely positioned using ultrasound guidance at the saphenofemoral junction heel and checked both before and after the infusion of tumescent anesthesia (Figure 3). In all cases, including those involving ASV with anatomically challenging trajectories, we were able to position the fiber flush to the SFJ. This was achieved through real-time manipulation of the fiber by gentle skin pressure and ultrasound-guided adjustments in vein alignment via tumescent infiltration.

Technical feasibility was scrutinized based on the precision in positioning and confirming the visibility of the catheter tip at the SFJ after administering tumescent anesthesia. Power settings were adjusted between 8 and 14 W as the laser fiber was retracted, in correlation with the diameter of the vein and the quality of the vein wall. The power level and the speed of fiber retraction were adjusted to the changes in echogenicity in the vein wall structure [5,20,21,22]. The power level was decreased stepwise if the fiber tip was sticking to the vessel wall, until the withdrawal movement was fluent. Fiber withdrawal was performed with a manual pull-back technique under continuous ultrasound, without an automatic retraction device, allowing instantaneous adjustment of retraction speed in response to local vein caliber, wall echogenicity, and early signs of dome–intima adhesion [19,22].

Post-ablation, adjunctive treatments such as multiple-stab phlebectomies or foam sclerotherapy (less than 10 mL of foam per session [23]) were performed to address residual above or below knee varicosities. Postoperative protocol included eccentric compression dressings, class I compression stockings, and a prophylactic dose of rivaroxaban (10 mg daily 3–5 days) for non-chronically anticoagulated patients, according to their VTE risk stratification, which included factors such as CEAP class, BMI, history of thrombosis, extent of immobilization, and extent of adjunctive procedures [24]. Patients were encouraged to ambulate immediately after the procedure, promoting a quick return to routine activities, with oral pain management medications (paracetamol and NSAIDs) indicated as required. Following the EVLA procedure, patients were advised to remove the compressive dressing after 48 h and to continue wearing class 1 compressive stockings for two weeks [9,19,25,26].

A one-week follow-up ultrasound examination was conducted by the attending physician with the patient in a standing position, using longitudinal and transverse views to assess the saphenofemoral junction and both deep and superficial venous systems [19,25]. This examination focused on evaluation of the relationship of the thrombus from the confluence of the superficial inguinal veins with the common femoral vein (CFV), classifying EHIT as per Kabnick et al.’s criteria [9]. In cases with EHIT 3 or 4, follow-up was also performed 4 weeks after the intervention, otherwise at 12 weeks, by doppler ultrasound [19,27,28].

Residual stump length was measured in the longitudinal view as the linear distance from the deepest point of the CFV confluence to the proximal margin of the obliterated segment, and reconfirmed transversely to minimize angular over- or under-estimation; consistent with contemporary consensus, EHIT classes I–II were recorded to document closure patterns but were interpreted primarily as occlusion states, whereas EHIT III–IV constituted the primary safety endpoint and prompted weekly surveillance until retraction or resolution [9].

Complications such as deep vein thrombosis, clinical signs of pulmonary embolism (PE), allergies, sensory disturbances, bleeding, and infection were recorded. Anatomic success was evaluated based on the GSV’s closure status, distinguishing between complete closure, partial closure, and recanalization in treated vein segments.

Normality of variables such as age, saphenofemoral junction diameter, vein diameter, BMI, and bilateral interventions was assessed using the Kolmogorov–Smirnov and Shapiro–Wilk tests. The Independent-Samples Kruskal–Wallis test was utilized to compare distributions of SFJ diameter, vein diameter, age, power setting, and venous stump length across different EHIT categories. Chi-square tests were used to examine associations between categorical variables, such as the relationship between the distribution of SFJ diameter and EHIT categories. Pearson correlation assessed the linear relationship between continuous variables like venous stump length and vein diameter, while Spearman correlation evaluated the monotonic relationships between variables that might not have a linear correlation.

## 3. Results

A total of 369 GSVs and 36 ASVs were subjected to flush ablation procedures in 312 patients (405 limbs). Ninety-four patients (30.12%) received bilateral ablations and in 86 of these cases (27.5%), interventions were performed on both limbs during the same procedure. Detailed classification of clinical characteristics per limb according to the CEAP criteria is provided in Table 2.

Across the spectrum of procedures, one case was excluded due to conversion to open ligation. Ultrasound imaging clearly visualized the junction and ablation catheter tip in all cases. Patient demographics are elaborated in Table 2. Junction diameters were observed at a median of 9.75 ± 3.06 mm, ranging from 3.5 to 27 mm, while vein diameter averaged 10.06 ± 5.54 mm, with a range of 1 to 30 mm. Post-tumescent anesthesia, catheter tips were accurately repositioned as needed, addressing any dislocations into the superficial epigastric vein or further along the treated vein. Parameters pertaining to the intervention are succinctly summarized in Table 3.

Except in one instance, adjunctive treatments were performed, including phlebectomies and ligations or laser ablation of the refluxing perforators (n = 323), or combined with intraoperative foam sclerotherapy (n = 81). Laser settings were adjusted according to patient-specific anatomical and pathological requirements, with power settings ranging from 5 to 14 watts, averaging 10.62 ± 0.17 watts, corroborated to intraoperative ultrasound appearance of the vein wall or blockage of withdrawal by catheter tip sticking to the vein wall.

One week follow-up DUS assessments of the saphenofemoral junction was conducted, with a combined success rate of 96.7% (n = 327 EHIT 1, n = 65 EHIT 2) in achieving closure (Table 4), albeit 25 legs (6.17%) exhibited residual saphenous stumps averaging 0.65 ± 0.26 mm (Table 5).

**Figure 4:**
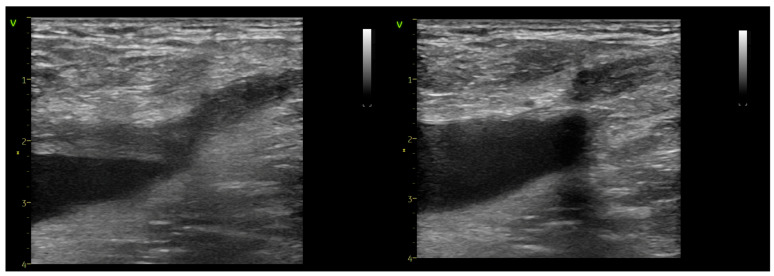
Follow-up imaging of an EHIT class 3 case at 7 days (**left**) and 21 days (**right**).

Non-symptomatic EHIT class 2 did not necessitate anticoagulant therapy. For those presenting with EHIT class 3 (n = 10; 2,5%) and 4 (n = 1; 0,2%) complications, continuation of compression stockings, rivaroxaban 15 mg twice daily for 21 days, and venous rehabilitation (lifestyle adaptation and physical therapies) were recommended [21]. Subsequent re-imaging confirmed thrombus resorption from the common femoral vein at one month, with no additional changes in thrombus extension observed during the following two months of monitoring (Figure 4).

Twenty-five legs presented with minor complications at one week and four exhibited hematomas ranging from 1.2 to 3 cm, all of which were directly related to phlebectomy sites and were fully resorbed at the 12-week follow-up. Three legs (0.74%) suffered recanalization of the treated vein, with two presenting a patent ASV with reflux, and one occurring 10 cm distally from the SFJ, over a length of 6 cm.

No cases of clinically suspected PE were observed during the study or follow-up period. At each follow-up visit, patients were systematically evaluated for signs and symptoms suggestive of PE, including sudden onset of dyspnea, chest pain, tachycardia, or unexplained anxiety. As no clinical suspicion of PE was raised, no CT pulmonary angiography was deemed necessary or performed.

Across EHIT categories, there were no significant differences in age (*p* = 0.234), SFJ diameter (*p* = 0.627), mid-thigh vein diameter (*p* = 0.440), or BMI (*p* = 0.510). By contrast, the generator power set at the SFJ was higher in limbs that developed EHIT III–IV (*p* = 0.004). This association remained significant after adjustment for BMI (as a continuous variable and at ≥30), treated side, operative time, CEAP class, and diameters (*p* < 0.05), while none of these covariates showed independent associations with EHIT III–IV (all *p* > 0.05).

Across the cohort, eleven limbs met criteria for clinically significant thrombus extension at the junction (ten EHIT III and one EHIT IV), typically identified at the protocolled one-week duplex assessment and uniformly managed with therapeutic anticoagulation and short-interval ultrasound surveillance until retraction below the SFJ. In the pre-specified analyses, no conventional patient-level covariate—including BMI as a continuous variable and BMI ≥ 30 as a categorical split, treated side (left vs. right), operative time, or clinical CEAP class—showed a statistically significant association with the occurrence of EHIT III–IV at the α = 0.05 threshold (all *p* > 0.05), indicating that the junctional thrombus propagation observed here was not explained by baseline anthropometrics, laterality, procedure duration, or clinical stage. Within the EHIT III subgroup, we observed a limited, non-significant clustering of elevated BMI: two of ten EHIT III limbs occurred in patients with BMI ≥ 30, but this distribution did not translate into a meaningful effect in either the continuous or dichotomized models (*p* > 0.05 for both contrasts), and no other patient- or limb-level covariate demonstrated a signal toward increased risk. By contrast, the only variable exhibiting a statistically significant association with EHIT III–IV was greater intra-procedural power delivery at the junction, operationalized in this dataset as a higher generator power setting (watts) at the SFJ; practically, this reflected the device’s W setting in combination with operator-modulated pull-back, such that higher W with slower withdrawal yielded greater cumulative power exposure at the heel of the SFJ, and this effect remained significant after adjustment for BMI, side, operative time, CEAP class, and other covariates (*p* < 0.05). Importantly, none of the 11 patients with EHIT III–IV had documented specific venous-thromboembolism risk factors beyond the procedural exposure—there were no known prothrombotic conditions recorded, no active oncologic disease, and no other predefined VTE triggers in the chart abstraction—which further reinforces the primacy of perijunctional power delivery rather than baseline thrombophilia in the pathogenesis of these events (all *p* > 0.05 for patient-level risk factors in univariable screens).

## 4. Discussion

The success rates reported in this study, characterized by adequate vein closure and minimal numbers of residual saphenous stumps (n = 25/405), align well with findings from Spinedi et al. (2021), who demonstrated a 94.1% technical feasibility of flush fEVLA, with flush occlusion rates maintaining over 88% effectiveness at a six-week follow-up [12]. In our cohort, we report higher flush occlusion rates of 96.7%. Similarly, studies by Hamann et al. [1] have highlighted the safe and effective application of fEVLA even in complex cases involving venous aneurysms near saphenofemoral junctions, reinforcing the adaptability and robustness of fEVLA techniques.

Our observed recanalization rate of 0.74% stands significantly lower than the rates typically reported in the existing literature. Notably, Theivacumar et al. have identified that suboptimal laser energy settings can lead to recanalization rates as high as 15% [29], underscoring the critical importance of adequate energy delivery for achieving sustainable vein closure. Similarly, Disselhoff et al. (2008) emphasize the necessity of precise catheter placement, attributing recanalization rates of ~10% [22] to inaccuracies in catheter positioning. The substantially lower recanalization rate in our study may indicate precision in both energy deployment and catheter positioning, suggesting that attention to these procedural details could be instrumental in enhancing the long-term efficacy of fEVLA.

A comprehensive analysis of the incidence and risk factors of EHIT after fFEVLA reveals significant variations across different studies. Kane et al. (2014) reported an EHIT incidence of 5.1% [30] in a cohort of 528 treated veins, identifying larger vein diameters as a critical risk factor, with EHIT typically resolving within one week under observation and anticoagulation therapy. In contrast, Sufian et al. (2015) observed a markedly lower incidence of 0.9% [31] in 2168 treated limbs, with older age and multiple phlebectomies also contributing to EHIT development, and resolution occurring over 2–4 weeks. Similarly, Ryer et al. (2016) reported a 5.1% incidence in 842 procedures, emphasizing the importance of post-procedure ultrasound for early detection, with nearly half of the cases detected on delayed scans [32]. Dermody et al. (2015) noted that EHIT incidence could reach up to 6.4% [33] depending on the method of fEVLA and patient characteristics (female sex, higher age), further highlighting the variability in reported rates. Donagh et al. (2018) focused on the risk factors associated with EHIT, such as increased saphenous vein diameter [34].

In a series of retrospective analyses, the diameter of the GSV has been identified as a significant predictor of EHIT. Sermsathanasawadi et al. (2016) demonstrated that a GSV diameter exceeding 10 mm significantly increases EHIT risk, with an odds ratio (OR) of 5.97 and a 95% confidence interval (CI) from 1.161 to 30.716 (*p* < 0.05) [35]. Similarly, Harlander-Locke et al. (2013) found that a GSV diameter greater than 8 mm (*p* = 0.027; 95% CI, 3.66–9.89) is associated with higher EHIT risk and a significant association between a history of previous DVT and the occurrence of EHIT in their analysis of 1000 vein ablations (*p* = 0.041) [36]. Rhee et al. [37] (2013) and Jacobs et al. (2013) identified the male sex as a significant risk factor for EHIT, with odds ratios of 5.98 (*p* = 0.0003) and 4.91 (*p* = 0.027), respectively [38].

Alozai et al. conducted a randomized, single-blind, controlled trial in patients with GSV/SFJ incompetence and a competent ASV, comparing standard EVLA with flush EVLA using a 1470 nm radial two-ring fiber, with ASV reflux prespecified as the primary endpoint and assessed by duplex at 1 week, 6, 12, and 24 months; 52 patients were randomized to standard EVLA and 49 to fEVLA, achieving near-identical truncal occlusion at 24 months (98% vs. 100%, *p* = 0.33), while flush positioning halved the mean stump length at the junction (4 ± 4 mm vs. 8 ± 4 mm, *p* < 0.001), yet did not reduce new-onset ASV reflux at two years (30% after fEVLA vs. 21% after standard EVLA; RR 1.53, 95% CI 0.64–3.66; *p* = 0.34; Kaplan–Meier log-rank χ^2^ = 0.68, *p* = 0.41) [15]. Secondary outcomes were broadly similar between groups, including pain, time to return to daily activities, cosmetic assessments, VCSS, AVVQ, reinterventions, and non-junctional complications; the principal junctional safety difference was a higher rate of EHIT I after fEVLA (57% vs. 17%, *p* < 0.001), with EHIT II uncommon and not different (2% vs. 6%, *p* = 0.34). Taken together, the trial shows that flush placement reliably shortens the residual stump but, in cohorts with a competent baseline ASV, this technical gain does not translate into less ASV reflux at mid-term follow-up, supporting a nuanced view that anatomy, energy delivery, and operator technique may condition whether flush positioning yields clinical advantages beyond anatomic neatness.

Bontinis et al. performed a PRISMA-guided systematic review and meta-analysis encompassing 11 studies and 3147 truncal veins in 2738 patients, synthesizing both single-arm fEVLA series and comparisons vs. standard EVLA, with EHIT ≥ II set as the primary pooled safety endpoint; the analysis found very low pooled EHIT ≥ II (1.37%, 95% CI 0.57–3.28), high medium-term GSV occlusion (97.59%, 95% CI 94.89–98.88), and rare thromboembolic events (DVT 0.97%, PE 0.04%), while meta-regression indicated that higher linear endovenous energy density (LEED) at the SFJ correlated with increased EHIT ≥ II risk (β = 0.011, *p* < 0.01), underscoring the importance of dosimetry at the junction in flush strategies [14]. In comparative subsets, fEVLA showed shorter stumps (MD −7.23 mm, 95% CI −11.59 to −2.88) and lower proximal groin recurrence (RR 0.35, 95% CI 0.16–0.80) versus standard EVLA, with non-significant trends favoring fEVLA for GSV occlusion (OR 3.26, 95% CI 0.76–13.97) and against new AASV reflux (RR 0.45, 95% CI 0.11–1.77). The authors conclude that fEVLA is safe and may confer anatomically plausible advantages at the SFJ (less residual stump, fewer groin-level recurrences) while acknowledging that higher-quality comparative trials are needed—a conclusion that aligns with the RCT, which demonstrates anatomic benefits without a clear reduction in AASV reflux at two years. Collectively, these data support presenting fEVLA as a technically feasible, low-EHIT approach whose clinical value likely depends on local anatomy and controlled energy delivery at the junction.

Our findings on the incidence and management of EHIT are consistent with the recent guidelines outlined by Tan et al. in their 2024 report [39]. The authors emphasize the use of the AVF-EHIT classification system to guide management, recommending different degrees of intervention based on the extent of thrombus propagation (Table 1). This aligns with our approach, which also focuses on tailored patient management. Specifically, we observed that individualized treatment strategies, including appropriate use of anticoagulation and rigorous post-procedural monitoring, are effective in reducing EHIT-related complications.

Our findings indicate that patient- and limb-level covariates—age, BMI (both continuous and ≥30), laterality, operative time, CEAP class, and junctional or truncal diameter—did not differ across EHIT categories (all *p* > 0.05), while the only independent association with EHIT III–IV had greater intra-procedural power at the junction, expressed as a higher generator power setting (watts) at the SFJ in combination, with slower operator-modulated pull-back (power term *p* < 0.05); notably, within EHIT III, we observed two out of ten cases with BMI ≥ 30, but BMI did not reach statistical significance, and none of the eleven EHIT III–IV patients carried chart-documented specific VTE risk factors, suggesting that perijunctional dosing (power and dwell) rather than baseline thrombophilia was the relevant driver in our series. This pattern aligns with the randomized EJVS trial, where flush positioning increased early EHIT I without a signal for excess clinically significant events and did not improve mid-term AASV reflux—an outcome that implicitly points to how much energy is delivered at the heel, not merely where the tip sits, as a key safety determinant [15]. It also sits comfortably with AVF/SVS guidance that emphasizes junction-focused risk mitigation and standardized EHIT management pathways [9]. Conversely, prior retrospective work has linked larger GSV diameters (e.g., >10 mm) and longer procedures to higher EHIT risk—associations we did not reproduce—possibly reflecting differences in technology (1470–1550 nm, radial fibers), tumescent technique, and contemporary ultrasound-guided tip control in our practice [35]. From a thrombosis-risk standpoint, the best-quality pooled evidence for flush EVLA reports high occlusion with low thrombotic event rates (EHIT ≥ II uncommon; DVT/PE rare), while also cautioning that greater junctional energy exposure associates with more severe EHIT—reinforcing the clinical relevance of power moderation and meticulous pull-back at the SFJ in routine care [6,14].

## 5. Limitations

This study analyzed 405 flush ablations from a single center, which may not generalize to broader populations. The 90-day follow-up period provides valuable initial insights, but longer-term outcomes and complication rates are needed. Because LEED and treated-segment length were not documented consistently in a sufficient proportion of cases, these variables were not incorporated into the study dataset for statistical analysis.

## 6. Conclusions

fEVLA is an effective and technically feasible treatment for chronic superficial venous insufficiency, with high rates of vein closure and minimal complications. The findings highlight the importance of precise procedural techniques, adequate power settings, and vigilant postoperative monitoring to mitigate risks such as EHIT. Despite limitations, this study confirms the efficacy and safety of fEVLA, suggesting potential for broader clinical application. However, long-term data are still needed.

## Figures and Tables

**Table 1:** AVF-EHIT classification system and treatment.

AVF-EHIT CLASS	Definition	Treatment Recommendation
I	Thrombus without propagation into deep vein a. Peripheral to superficial epigastric vein b. Central to superficial epigastric vein, up to and including the deep vein junction	No treatment or surveillance.
II	Thrombus propagation into the adjacent deep vein, but comprising < 50% of the deep vein lumen	No treatment, weekly surveillance until thrombus resolution. In high-risk patients consider antiplatelet therapy vs. anticoagulation. Discontinue treatment following thrombus retraction or resolution.
III	Thrombus propagation into the adjacent deep vein, but comprising > 50% of the deep vein lumen	Therapeutic anticoagulation, weekly surveillance. Discontinue treatment following thrombus retraction or resolution.
IV	Occlusive deep vein thrombosis contiguous with the treated superficial vein	Treatment should be individualized, taking into account risks and benefits to patient.

**Table 2:** CEAP classification for enrolled patients.

CEAP Classification	Number of Limbs
1	1
2	24
3	147
4	164
5	13
6	56

**Table 3:** Interventional parameters.

Variable	Mean ± SD	Range
Age	53.27 ± 13.28	21.00–92.00
SFJ Diameter (mm)	9.75 ± 3.06	3.50–27.00
Vein Diameter (mm) (Mid Tigh)	10.06 ± 5.54	1.00–30.00
BMI (kg/m^2^)	27.90 ± 3.93	19.03–42.17
Power (W)	10.62 ± 1.71	5.00–14.00

**Table 4:** EHIT classification for enrolled patient at one week follow-up.

EHIT Classification	Frequency	Percent
1	327	80.7%
2	65	16.0%
3	10	2.5%
4	1	0.2%
Total	404	99.5%
Missing	1	0.5%
Total	405	100.0%

**Table 5:** Residual venous stump length at one week follow-up.

Venous Stump Length (mm)	Frequency	Percent
0	380	93.8%
6	3	0.5%
7	1	0.2%
8	1	0.2%
9	4	1.0%
10	4	1.0%
11	2	0.5%
12	3	0.7%
13	4	1.0%
15	2	0.5%
16	1	0.2%
Total	405	100.0%

## Data Availability

The original contributions presented in this study are included in the article. Further inquiries can be directed to the corresponding author(s).
